# Genomic Epidemiology and Multilevel Genome Typing of Australian Salmonella enterica Serovar Enteritidis

**DOI:** 10.1128/spectrum.03014-22

**Published:** 2023-01-10

**Authors:** Lijuan Luo, Michael Payne, Qinning Wang, Sandeep Kaur, Irani U. Rathnayake, Rikki Graham, Mailie Gall, Jenny Draper, Elena Martinez, Sophie Octavia, Mark M. Tanaka, Amy V. Jennison, Vitali Sintchenko, Ruiting Lan

**Affiliations:** a School of Biotechnology and Biomolecular Sciences, University of New South Wales, Sydney, New South Wales, Australia; b Centre for Infectious Diseases and Microbiology–Public Health, Institute of Clinical Pathology and Medical Research–NSW Health Pathology, Westmead Hospital, Westmead, New South Wales, Australia; c Public Health Microbiology, Forensic and Scientific Services, Queensland Department of Health, Coopers Plains, Queensland, Australia; d Sydney Institute for Infectious Diseases, Sydney Medical School, University of Sydney, Sydney, New South Wales, Australia; The Pennsylvania State University

**Keywords:** *Salmonella* Enteritidis, genomic epidemiology, foodborne outbreak, multilevel genome typing, standardized genomic typing, genomic typing database

## Abstract

Salmonella enterica serovar Enteritidis is one of the leading causes of salmonellosis in Australia. In this study, a total of 568 *S.* Enteritidis isolates from two Australian states across two consecutive years were analyzed and compared to international strains, using the *S.* Enteritidis multilevel genome typing (MGT) database, which contained 40,390 publicly available genomes from 99 countries. The Australian *S.* Enteritidis isolates were divided into three phylogenetic clades (A, B, and C). Clades A and C represented 16.4% and 3.5% of the total isolates, respectively, and were of local origin. Clade B accounted for 80.1% of the isolates which belonged to seven previously defined lineages but was dominated by the global epidemic lineage. At the MGT5 level, three out of five top sequence types (STs) in Australia were also top STs in Asia, suggesting that a fair proportion of Australian *S.* Enteritidis cases may be epidemiologically linked with Asian strains. In 2018, a large egg-associated local outbreak was caused by a recently defined clade B lineage prevalent in Europe and was closely related, but not directly linked, to three European isolates. Additionally, over half (54.8%) of predicted multidrug resistance (MDR) isolates belonged to 10 MDR-associated MGT-STs, which were also frequent in Asian *S.* Enteritidis . Overall, this study investigated the genomic epidemiology of *S.* Enteritidis in Australia, including the first large local outbreak, using MGT. The open MGT platform enables a standardized and sharable nomenclature that can be effectively applied to public health for unified surveillance of *S.* Enteritidis nationally and globally.

**IMPORTANCE** Salmonella enterica serovar Enteritidis is a leading cause of foodborne infections. We previously developed a genomic typing database (MGTdb) for *S.* Enteritidis to facilitate global surveillance of this pathogen. In this study, we examined the genomic features of Australian *S.* Enteritidis using the MGTdb and found that Australian *S.* Enteritidis is mainly epidemiologically linked with Asian strains (especially strains carrying antimicrobial resistance genes), followed by European strains. The first large-scale egg-associated local outbreak in Australia was caused by a recently defined lineage prevalent in Europe, and three European isolates in the MGTdb were closely related but not directly linked to this outbreak. In summary, the *S.* Enteritidis MGTdb open platform is shown to be a potentially powerful tool for national and global public health surveillance of this pathogen.

## INTRODUCTION

Salmonella enterica serovar Enteritidis is one of the most prevalent Salmonella serovars causing foodborne infections (1). *S.* Enteritidis mainly causes gastroenteritis but can also lead to nontyphoidal invasive salmonellosis (iNTS) in humans (2). *S.* Enteritidis is widely distributed across different zoonotic niches and is especially common in poultry and poultry products such as eggs (3). The prevalence of *S.* Enteritidis increased substantially in the late 20th century in Europe and North America (4). In many countries (e.g., United States, United Kingdom, Uruguay, Lebanon), *S.* Enteritidis is ranked as the most common serovar responsible for human infections (5–8). In the past few years, large-scale multinational outbreaks of *S.* Enteritidis were reported (9–13). From 2017 to 2020, a multistate outbreak of *S.* Enteritidis in Europe resulted in 656 confirmed cases (14).

Based on genome-wide comparisons, *S.* Enteritidis has been divided into three clades, A, B, and C, by Graham et al. (15), and five HierCC clusters by Achtman et al. (16). Clade B is the predominant clade, which was further divided into 10 lineages in our previous study (17). These clades and lineages have different geographical distributions (17). Clades A and C were prevalent in Oceania but were relatively rare in other continents (17). Within clade B, two dominant lineages represented more than 85% of clade B genomes and had different geographic distributions, outbreak propensities, and evolutionary characteristics (17). In addition to these two dominant lineages, two African lineages within clade B were associated with iNTS (mainly in sub-Saharan African low-income countries) and multidrug resistance (MDR) (2).

In Australia, S. Enteritidis is the second most prevalent serovar (18–20). Clade A and clade C *S.* Enteritidis were previously documented in the state of Queensland (QLD) (15), while their distribution in other Australian states remained unknown. Clade B isolates in Australia were considered imported from other countries by travelers (15) and were rarely linked to local outbreaks (15, 21, 22). However, a local egg-associated outbreak of clade B *S.* Enteritidis (from 2018 to 2019) caused more than 200 cases (23, 24). In addition, antimicrobial resistance (AMR) genes were rarely present in the local clade A and C strains but had high frequency in clade B genomes (15). As *S.* Enteritidis has become an increasing threat in Australia, comparing the genomic features of Australian *S.* Enteritidis with international strains would help in understanding the source of Australian *S.* Enteritidis.

**FIG 1:**
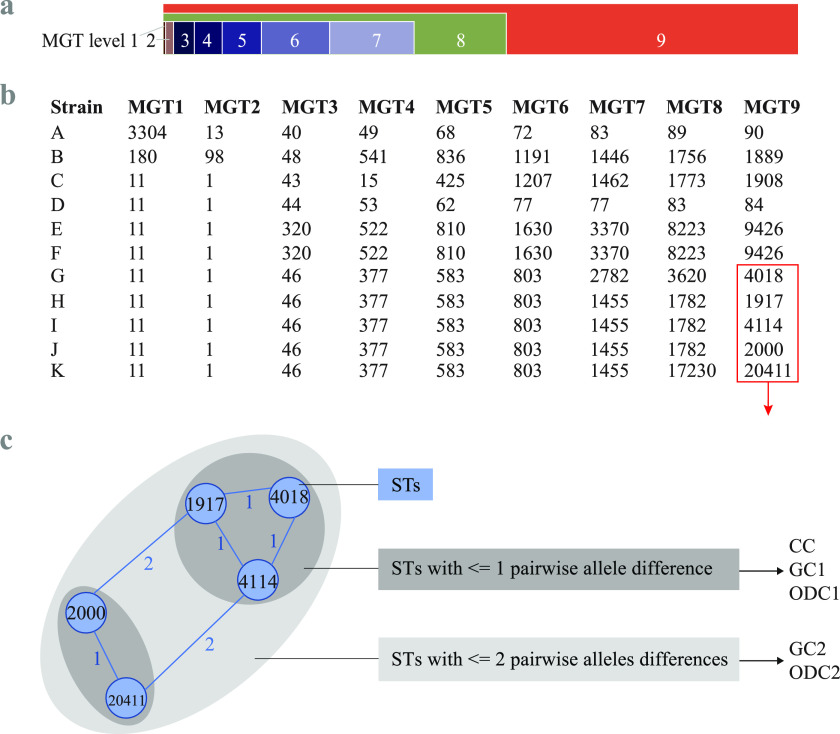
Schematics of MGT and definition of single-linkage clusters. (a) MGT scheme of 9 levels with an increasing number of loci from MGT1 (the classic 7-gene MLST scheme) to MGT9 (the cgMLST scheme of *S.* Enteritidis with 4,986 loci). MGT1 to MGT7 were exclusive from each other with no shared loci. MGT8 and MGT9 included loci of the lower levels. (Adapted from reference 25.) (b) ST calling at different MGT levels. For each strain, an ST was assigned based on exact comparison and a unique combination of alleles at each MGT level. (c) Single-linkage clustering of STs using different cutoffs. For all MGT levels, clonal complex (CC) is defined as STs with a one-allele difference. At MGT9, outbreak detection clusters (ODC) or genomic clusters (GC) are defined as follows: ODC1 (GC1, also MGT9 CC), ODC2 (GC2), ODC5 (GC5), and ODC10 (GC10), allowing for pairwise allele differences of 1, 2, 5, and 10, respectively.

A unified global genomic surveillance system would contribute to tracking and controlling the spread of outbreak clusters and MDR clones in Australia. Multilevel genome typing (MGT) for *S.* Enteritidis (17) is a recently defined and publicly accessible whole-genome sequencing (WGS) typing scheme (https://mgtdb.unsw.edu.au/enteritidis/). The key benefit of MGT is the provision of multiple-resolution typing levels with sequence types (STs) assigned at each level (25) (Fig. 1). The exact comparison-based STs can avoid the drawbacks of clusters merging using the single-linkage clustering algorithm, which enhances the stability of the ST assignments (25). The STs from different resolution levels have been found useful in investigating the epidemiological distribution of *S.* Enteritidis and tracking MDR clones (17). Moreover, the serovar core genome multilocus sequence typing (cgMLST) level MGT9, which includes the serovar core genes and core intergenic regions of *S*. Enteritidis, offered the highest resolution of typing for outbreak investigations (17, 26). Outbreak detection clusters (ODCs) are single-linkage clusters calculated based on MGT9, allowing a different number of allele differences (25). ODC1 (or MGT9 clonal complex [CC]), ODC2, ODC5, and ODC10 refer to single-linkage clustering with 1-, 2-, 5-, and 10-allele differences, respectively (25) (Fig. 1). These clustering schemes with different thresholds are built into the *S.* Enteritidis MGT database (MGTdb) and, together with metadata, allow the selection of appropriate cutoffs for optimal outbreak detection (17).

The global *S.* Enteritidis MGTdb (17) processes new publicly available genomes and metadata regularly, making it useful for national and international surveillance of *S.* Enteritidis. In this study, 568 newly sequenced genomes from two states of Australia, Queensland (QLD) and New South Wales (NSW), were investigated using *S.* Enteritidis MGT. The genomic characteristics of *S.* Enteritidis from Australia and other countries were compared to assess potential Australian *S.* Enteritidis outbreak clusters and to examine the application of MGT in the national surveillance of *S.* Enteritidis using the MGTdb.

## RESULTS

### Epidemiological characteristics of the sampled isolates.

**FIG 2:**
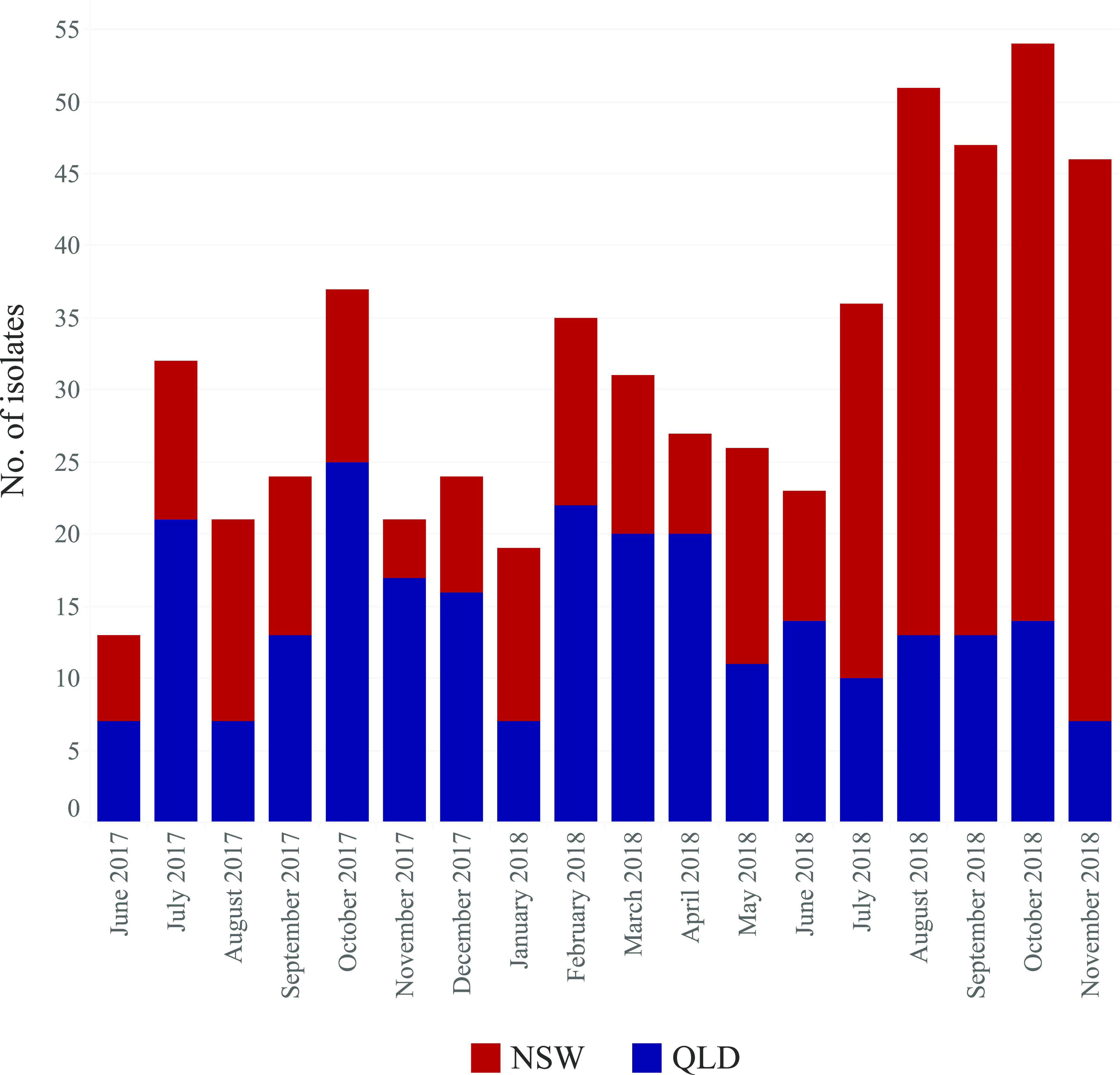
The collection dates of the newly sequenced genomes from NSW and QLD in Australia. The *x* axis shows the month and the year of collection, while *y* axis shows the number of isolates for each month.

A total of 896 Australian *S.* Enteritidis isolates were analyzed, of which 568 were newly sequenced genomes from two Australian states, QLD (257) and NSW (311), and the remainder (328) were publicly available genomes (Table 1). The newly sequenced isolates were collected from June 2017 to November 2018 (Fig. 2), except for one isolate, which was collected in July 2019. The collection period of all Australian isolates (including publicly available ones) was 2001 to July 2019.

**TABLE 1:** *S.* Enteritidis genome sequences used in this study

Isolate source	No. of isolates from:				Total isolates
	Australia			Other countries	
	QLD	NSW	Other states		
Newly sequenced	257	311			568
Publicly available	152	1	175	40,062	40,390
Total	409	312	175	40,062	40,958

The newly sequenced Australian *S.* Enteritidis genomes were compared to the updated *S.* Enteritidis MGTdb with 40,390 publicly available genomes which were collected from 99 different countries from 1917 to 2021. The detailed information of these genomes is described in the supplemental text, Fig. S1, and Table S1 in the supplemental material.

### MGT typing of newly sequenced isolates.

**FIG 3:**
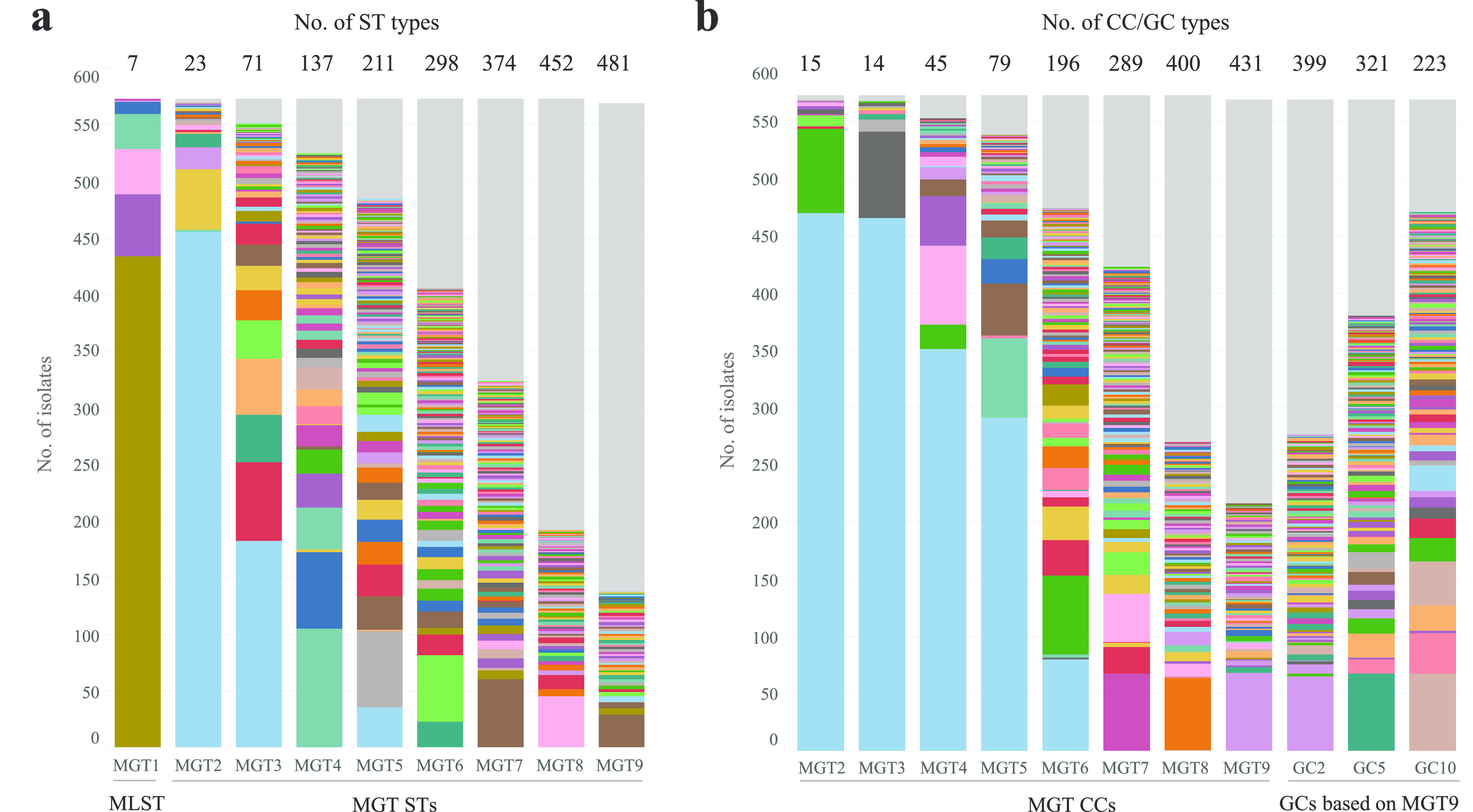
MGT typing of the newly sequenced genomes. (a) Sequence types (STs) assigned at each MGT level. (b) Single-linkage clustering of STs into clonal complexes (CCs) (assigned based on STs from MGT2 to MGT9) and genomic clusters (GCs) (assigned based on MGT9-STs). STs/CCs/GCs with ≥2 isolates each were separated into different colors, and singleton types (one isolate each) were grouped in gray.

The 568 newly sequenced Australian *S.* Enteritidis genomes were processed through the *S.* Enteritidis MGTdb and held as private data. STs were called from MGT2 to MGT9. MGT1 refers to the classic seven-gene MLST (1), which typed 99.7% of the 568 genomes into five STs. ST11 (78.9%) was the predominant type, followed by ST3304 (9.5%), ST1925 (7.0%), ST180 (5.3%), and ST1972 (1.9%). With the increasing resolution of typing from MGT2 to MGT9, there was an increasing number of STs called from 23 STs at MGT2 to 482 at MGT9 (Fig. 3a). Note that four genomes (0.7%, 4/568) failed to be called at the MGT9 level due to 2.2% to 2.6% alleles missing.

From MGT2 to MGT9, STs were grouped into CCs using the single-linkage clustering algorithm with a one-allele difference as the cutoff (Fig. 3b), using the same approach and definition as classic MLST (27). MGT9 STs were further grouped using the single-linkage clustering algorithm with more allele differences allowed (i.e., 2-, 5-, and 10-allele differences, which were used to generate genomic cluster 2 [GC2], GC5, and GC10, respectively) as described previously (Fig. 3b) (17, 25).

### Population structure of Australian S. Enteritidis isolates by clades and lineages.

**FIG 4:**
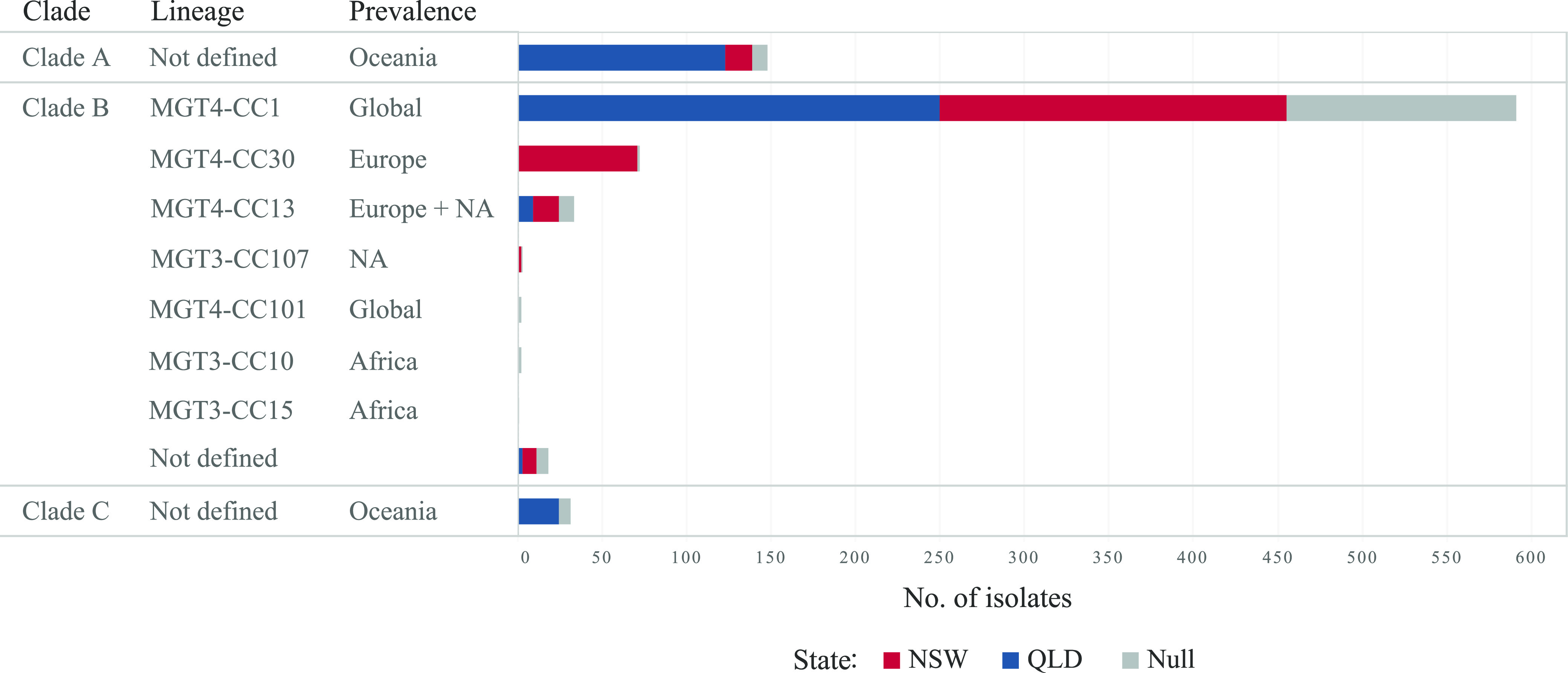
The population structure of the Australian *S.* Enteritidis using existing nomenclatures. NA, North America. The population structure of all Australian isolates (896), including the publicly available genomes. Seven previously defined lineages of clade B were observed, with the majority of isolates belonging to the global lineage MGT4-CC1. The two African lineages were observed with three isolates. Note that the geographic prevalence of different clades and lineages was based on the metadata in MGTdb, which may have sampling bias. The total number of isolates by state was marked with colour. Total with unknown origin (Null) was marked gray.

The population structure of *S.* Enteritidis was previously defined with three clades (A, B, and C) and 10 lineages within clade B (15, 17). Australian isolates fell into these clades and lineages. By clades, 16.4% (147), 80.1% (718), and 3.5% (31) of the 896 Australian *S.* Enteritidis genomes belonged to clade A, B, and C, respectively (Fig. 4). Within clade B, 7 of the previously defined 10 lineages were observed in Australia, representing 97.5% (700/718) of the clade B genomes (Fig. 4). The global lineage MGT4-CC1 was the predominant lineage (81.8%, 587/718), followed by the European lineage MGT4-CC30 (10.0%, 72/718) (Fig. 4). MGT4-CC13, which is especially prevalent in North America and Europe (17), was relatively rare in Australia (4.6%, 33/718). Three isolates belonged to the African endemic iNTS-associated lineages MGT3-CC10 (two MGT3-ST365 isolates) and CC15 (one MGT3-ST15 isolate) and were cultured from blood, wound, and ankle fluid samples, respectively. Only 18 clade B isolates (2.5%, 18/718) belonged to none of the 10 reported lineages.

### Comparison of Australian isolates across states and with international isolates using MGT5.

**FIG 5:**
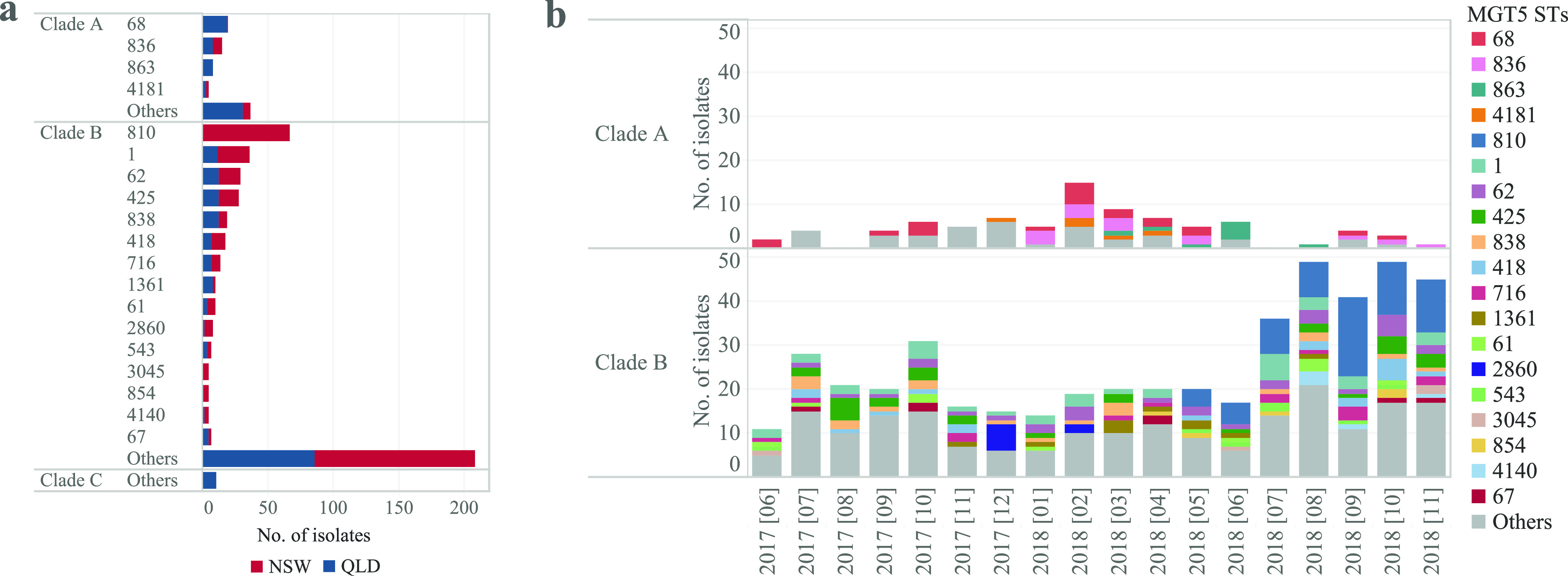
Genomic epidemiological characteristics of the MGT5-STs in Australia. (a) The main MGT5-STs (≥5 isolates each) of the newly sequenced Australian *S.* Enteritidis genomes, and their distribution by states (NSW and QLD). (b) Distribution of the main MGT5-STs in different months. STs with ≥5 isolates each are represented with different colors. Some STs occurred almost every month, whereas several STs appeared and persisted over several months.

The genomic epidemiology can be described at different MGT levels. In our previous study, we found that MGT5 described well the genomic epidemiology within the United Kingdom and United States (17). We also found that internationally circulating MGT4 STs can be further divided into country-specific/predominant STs at the MGT5 level. Hence, we used MGT5 to depict the Australian national genomic epidemiology of *S.* Enteritidis. We observed that there were 19 MGT5-STs with ≥5 isolates each, representing 55.6% of the genomes (Fig. 5). For clade C, the 11 isolates were sporadically observed during 7 months with no STs of more than five isolates each.

In clade A, there were four MGT5-STs with ≥5 isolates each, all of which were only observed in Australia. Three STs were observed in both QLD and NSW (MGT5-ST68, ST836, ST4181), and one ST (MGT5-ST863) was only observed in QLD (Fig. 5a). Across the 18-month sampling window from June 2017 to October 2018, MGT5-ST68 was observed over 10 months, MGT5-ST836 and MGT5-ST863 were observed over 7 and 5 months respectively, while other STs were observed over shorter periods (Fig. 5b). The top three STs, MGT5-ST68, ST836, and ST863, were found in the publicly available genomes from Australia in the MGTdb, whereas ST4181 was a newly identified type (Table 2). MGT5-ST68 ranked as the top ST and was first observed in 2005 in QLD, while the other three STs were observed from 2016 onward (Table 2).

**TABLE 2:** Comparison of the newly sequenced genomes with genomes in the *S.* Enteritidis MGTdb by MGT5-STs

No. of MGT5 STs	Clade	No. of MLST STs	Clade B MGT4	No. of new isolates	*S.* Enteritidis MGTdb^*a*^										
					No. of total isolates	Oceania^*b*^	Asia	Europe	North America	South America	Africa	No. of countries	Top country	Earliest yr	Invasive (%)
68	A	3,304		20	38	Top 4						1		2005	
836	A	180		14	17	+						1		2016	
863	A	3,304		8	9	+						1		2017	
4,181	A	3,304		5	5							1		2017	
810	B	11	CC30	67	97	+		+	+			7	Aus	1981	
1	B	11	CC1	36	2353	Top 1	Top 1	Top 1	Top 17	Top 1	Top 2	39	UK	1981	2.5
62	B	11	CC1	30	92	Top 2		+	+			4	Aus	2013	
425	B	11/1,925	CC1	28	167	Top 3	Top 5	+	+			8	UK	2008	1.2
838	B	11	CC1	19	53	+		+	+			4	UK	2015	
418	B	11	CC1	18	64	Top 5	+	+	+			4	Aus	2015	
716	B	11	CC1	14	65			+	+			5	UK	2016	
1,361	B	11	CC1	10	17	+		+	+			3	Aus	2017	
61	B	1,925	CC1	10	98	Top 6	Top 3	+	+			5	UK	2012	1.0
2,860	B	1,925	CC1	8	10			+				2	Aus	2017	
543	B	11	CC1	7	56	+	+	+	+			5	UK	2014	3.6
3,045	B	11	CC1	5	7			+				2	Aus	2017	
854	B	11	CC1	5	12	+		+				2	Aus/UK	2016	
4,140	B	11	CC1	5	5							1		2018	
67	B	11	CC13	7	201	+		Top 19	+		Top 4	8	UK	2004	6.5

a The top 20 STs are labeled; only STs with ≥5 isolates each were included for ranking. +, the continent containing the ST but it ranked outside of the top 20 STs.

b Publicly available genomes from Oceania with 99% from Australia.

In clade B, there were 15 MGT5-STs with ≥5 isolates; 86.7% (13/15) of the STs were found in both states (Fig. 5a). The 473 newly sequenced Australian clade B genomes were collected over 18 months (Fig. 5b). MGT5-ST1, ST62, ST425, ST838, and ST418 occurred almost every month. However, several STs were not continuously observed but, instead, appeared and persisted over several months. These STs included MGT5-ST810, emerged in May 2018, and MGT5-ST2860, emerged in December 2017. Both STs were observed with 5 or more isolates in 1 month.

By comparing the 15 main MGT5-STs of clade B in Australia with the publicly available genomes in the MGTdb, 14 (93.3%, 14/15) STs were also observed in other countries (Table 2), the dominant country of which was either Australia or the United Kingdom. MGT5-ST1 is the most frequently ST observed in multiple continents, including Oceania, Asia, Europe, and South America, and the second most frequent ST in Africa. Despite limited sampling from Asia (693 genomes in total in the MGTdb), five Australian STs were found in Asia, with three STs being ranked as the top, third, and fifth most common STs in Asian isolates (Table 2). In contrast, the majority of the clade B STs that were also found in Europe and North America were rarely ranked as the top STs in these places (except for MGT5-ST1). In terms of collection time, MGT5-ST810 and MGT5-ST1 were first sampled in 1981, while the other STs were first sampled in 2013. In addition, five Australian STs contained isolates that were collected from blood or cerebrospinal fluid (1.0% to 6.5% of the isolates for each ST), suggesting that these STs have caused invasive human infections.

### Using a 4-week window to identify potential outbreak clusters by different resolution levels of GCs.

MGT9, which included 4,986 loci, offered the highest resolution to identify closely related isolates. We further used genomic cluster (GCs) which are single-linkage clusters based on the MGT9 alleles to examine closely related clusters of isolates that were found across states and countries. GC1 (i.e., MGT9 CC), GC2, GC5, and GC10 corresponded to allele difference cutoffs of 1, 2, 5, and 10, respectively, for clustering MGT9 STs. Note that GCs are purely an alternative method of grouping isolates at small genetic distances. Using GCs solely without any other metadata cannot define outbreaks. Using the MGTdb, we compared the GC clusters across countries and states (NSW and QLD) as introduced in supplemental text and Fig. S2 and S3.

**FIG 6:**
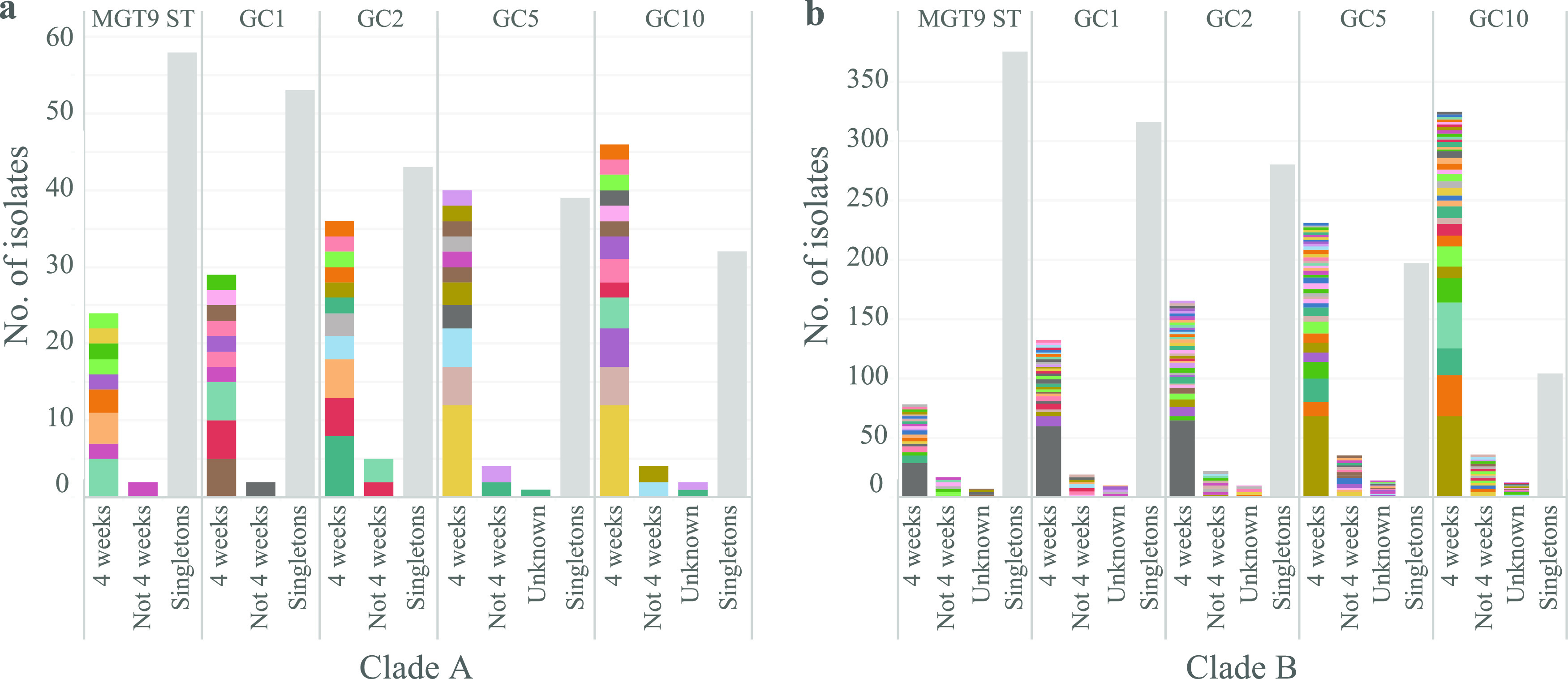
Identification of putative outbreak clusters using a 4-week window. Clusters with at least two isolates occurring within 4 weeks in clade A and B were identified at different-resolution genomic cluster (GC) levels. Clusters at each GC level were categorized into four groups. Singletons were clusters with only one isolate each, which were collapsed into one column in gray. For the nonsingleton clusters: 4 weeks, clusters including at least two isolates collected within 4 weeks; not 4 weeks, clusters that did not include two isolates collected within 4 weeks; unknown, clusters without collection date information.

For Salmonella outbreak investigation in NSW using multilocus variable-number tandem repeat analysis (MLVA), Sintchenko et al. defined a cluster of 5 cases with a specific MLVA type within a 4-week window as a threshold signaling an outbreak (28). However, for WGS data, no threshold of genome differences or minimum number of cases has been defined. We used a 4-week window and a minimum of 2 cases as a putative cluster definition of relevance to public health surveillance in this study. At different GC levels, the GC clusters that contained at least two isolates within 4 weeks (i.e., the minimum pairwise collection date distance of isolates was ≤4 weeks) were illustrated (Fig. 6). The clusters identified may also include isolates more than 4 weeks apart. Clusters with no collection date information were grouped as unknown. No epidemiological investigation metadata for the newly sequenced genomes were available to confirm whether any isolates were outbreak related except for the large outbreak in NSW described below.

For newly sequenced clade A genomes (84 isolates), there were 9, 10, 11, 11, and 13 clusters including isolates collected within 4 weeks at MGT9-ST, GC1, 2, 5, and 10, respectively (Fig. 6a). These clusters represented 28.6%, 34.5%, 42.9%, 46.4%, and 53.6% of the total 84 newly sequenced clade A genomes at MGT9-ST, GC1, 2, 5, and 10, respectively. For clade B, there were 20, 28, 38, 37, and 35 clusters including isolates collected within a 4-week window at MGT9-ST, GC1, 2, 5, and 10, respectively (Fig. 6b). These clusters accounted for 16.5%, 27.5%, 34.7%, 48.2%, and 67.9% of the 473 newly sequenced clade B genomes (from NSW and QLD) at MGT9-ST, GC1, 2, 5, and 10, respectively. For clade C, only one cluster was collected in 4 weeks at GC5 and GC10.

### Evolutionary analysis of the first Australian clade B *S.* Enteritidis outbreak of local origin.

A confirmed large-scale outbreak of clade B *S.* Enteritidis occurred in Australia in 2018 and lasted until 2019 and was the first known *S.* Enteritidis outbreak to have originated in a local farm source (23, 24). This outbreak was associated with contaminated eggs in NSW and caused more than 200 confirmed cases (23, 24). The majority of the cases were in NSW, but some were also observed in other states of Australia and in New Zealand (23, 24). The present study analyzed 68 genomes of outbreak isolates from NSW collected in 2018.

Different GC levels were examined to identify the level that best described the outbreak. By GC10, one cluster contained all Australian outbreak isolates but also contained 10 international isolates. By GC5, the number of international isolates was reduced to three, with two isolates from the United Kingdom and one from Ireland, isolates which were isolated from 2012 to 2015. By GC4, only the NSW isolates were clustered together without any international isolates. The NSW isolates mainly belonged to MGT7-ST3370 (90.0%).

**FIG 7:**
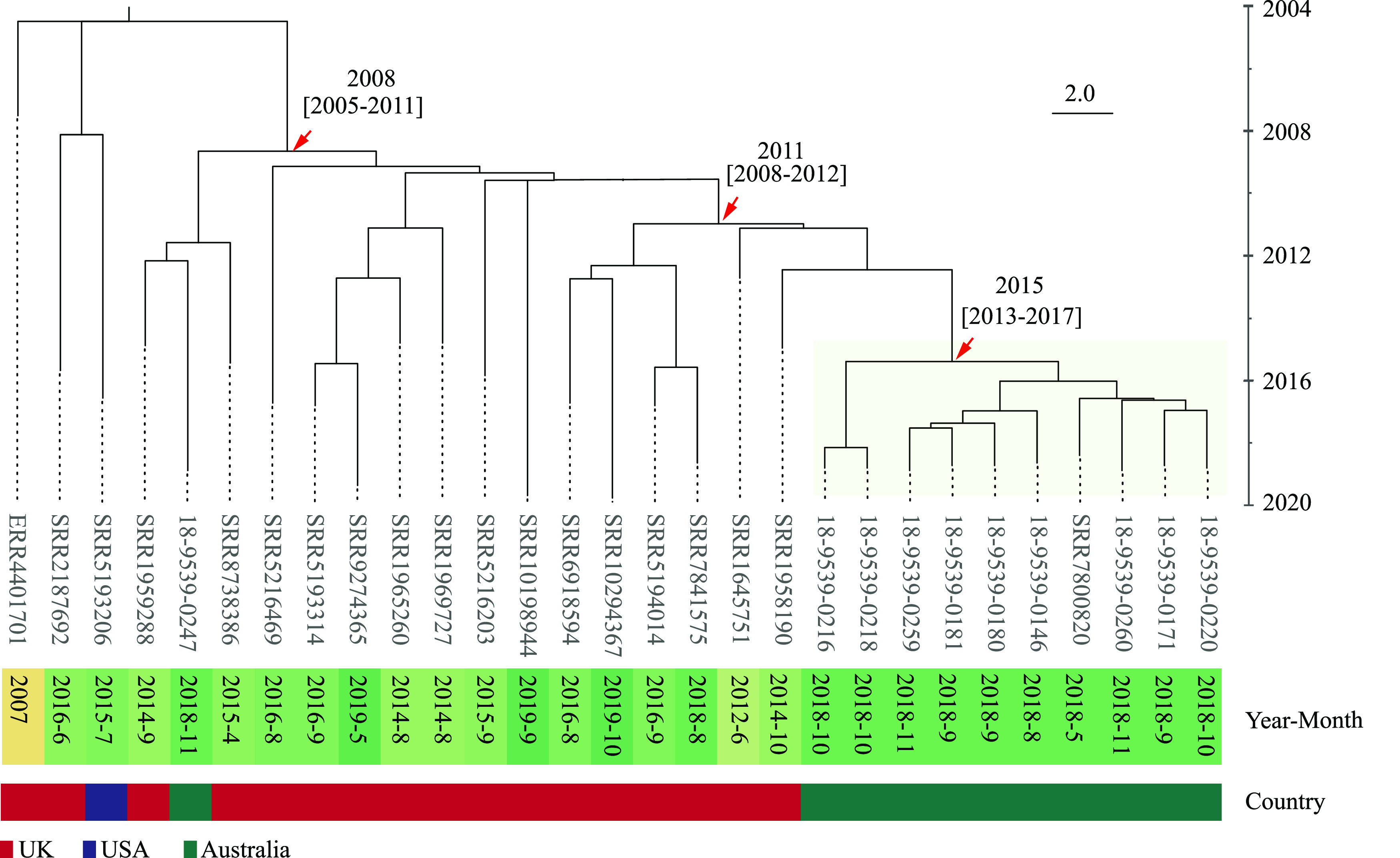
Phylodynamic analysis of the NSW outbreak-related isolates. A total of 29 isolates were sampled with the exponential growth strict clock model as the optimal model. Country and collection year-month information of the sampled isolates is represented in different colors. Isolates belonging to the Australian outbreaks are highlighted in green. The most recent common ancestor (MRCA) of the Australian outbreak isolates was around 2015 (95% CI, 2013 to 2017). Six isolates from the United Kingdom were phylogenetically close to the Australian outbreak isolates, and the MRCA was around 2011 (95% CI, 2008 to 2012). Note that one Australian isolate from 2018 that was unrelated to the large outbreak was identified and included in the tree.

To determine the evolutionary origins of the local *S.* Enteritidis lineage, we randomly sampled 29 genomes from the European common lineage MGT4-CC30 to which the Australian outbreak lineage belonged. Exponential growth and strict molecular clocks were evaluated as the optimal combination of settings in BEAST with a mean effective sample size (ESS) greater than 200. The most recent common ancestor (MRCA) of the Australian outbreak isolates was estimated to be around 2015 (95% confidence interval [CI], 2013 to 2017) (Fig. 7, green background). The MRCA of the Australian outbreak isolates and the UK isolates, which were the most closely related sampled isolates with the Australian outbreak, was estimated to be around 2011 (95% CI, 2008 to 2012) (Fig. 7). The Bayesian phylodynamic and population expansion of this lineage are shown in Fig. S4.

### Using Microreact to visualize and investigate large international outbreak clusters spread to Australia.

**FIG 8:**
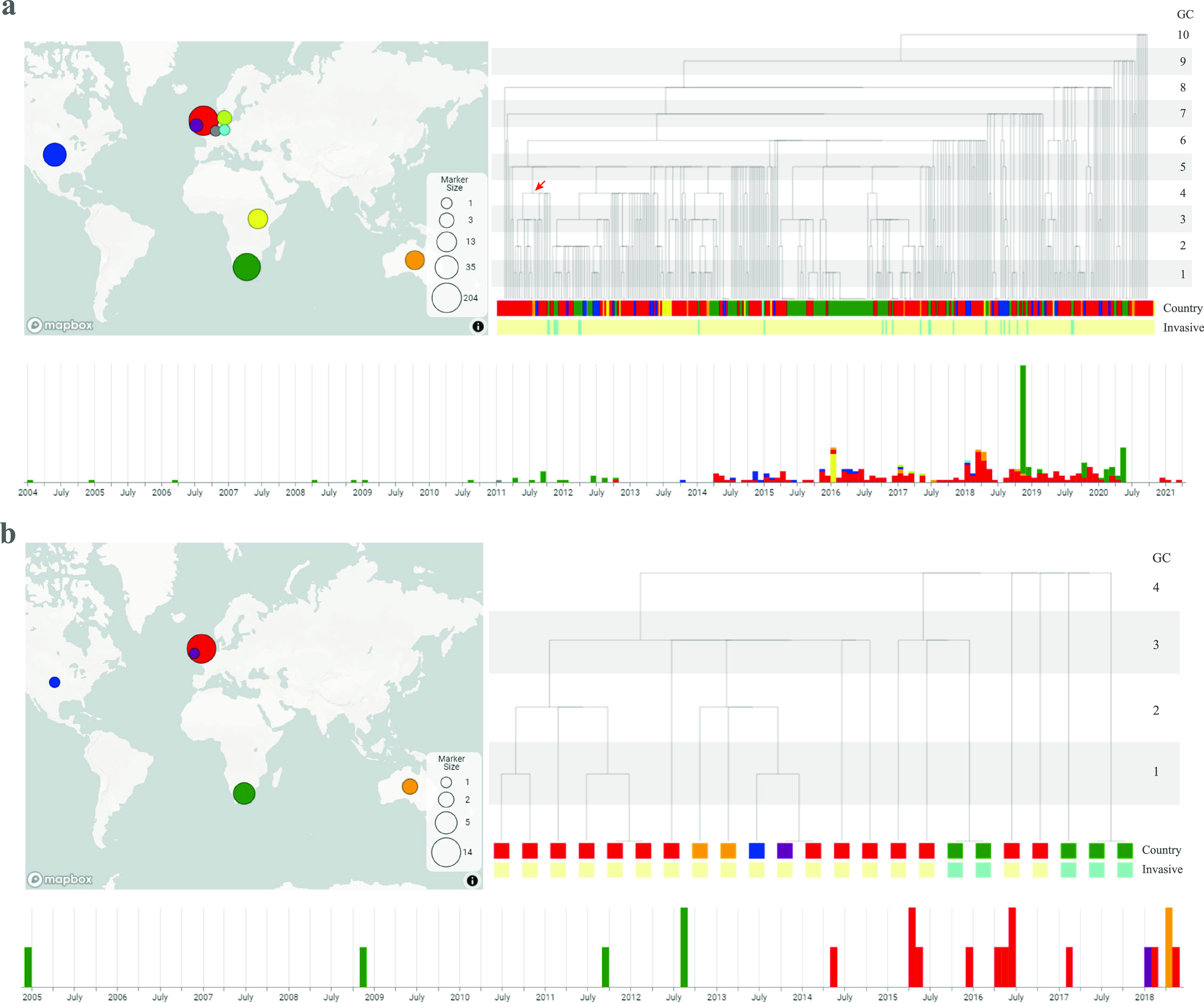
Visualization of an international cluster (GC10-C89) in Microreact. (a) Visualization of the whole cluster. The dendrogram was generated based on the cluster types from different resolution levels (MGT9 STs to GC10). The root is the GC10 cluster, and each leaf is an MGT9 ST. Each internal node is a GC between GC1 and GC9 as labeled. At each GC level, the branches that are linked with a horizontal line belong to the same cluster type. Different colors represent different countries. Isolates that caused possible invasive infections are labeled in green. The size of the circles in the map corresponds to the number of isolates. Note that international isolates with collection year and month information but no date were assumed to be collected on the 15th of each month. Subclusters at different GC levels can be selected and visualized. (b) Visualization of a GC4 subcluster (marked by the red arrow in panel a) as a separate tree. The two Australian S. Enteritidis genomes collected in 2018 were of the same GC2 type as three isolates from the United Kingdom, United States, and Ireland. The UK and Irish isolates were also observed in 2018.

International outbreak-related clusters have been observed in Australia. To visualize the temporal and spatial distribution of a single international cluster that includes Australian isolates, Microreact (29) in combination with a GC dendrogram was used. To investigate the relationship between the Australian isolates and international isolates and to demonstrate the usefulness of this approach, one large international cluster, GC10-cluster 89 (C89), was examined (Fig. 8a). GC10-C89, which was observed from 2004 to 2021, and comprised 392 isolates from 9 countries, including 11 isolates from Australia. Of the 11 Australian isolates, 6 belonged to 3 GC2 clusters (2 isolates each) collected within a 4-week window, suggesting potential small outbreak clusters. The leaves of the dendrogram represented isolates with different MGT9-STs; nodes represented subclusters from GC1 to GC9 within GC10-C89, which can be selected and visualized as a subtree (Fig. 8a). An international subcluster with 24 isolates was defined by a GC4 cluster (GC4-C5) (Fig. 8b). This GC4-C5 cluster was observed in Australia, the United Kingdom, the United States, South Africa, and Ireland from 2004 to 2018. Breaking this cluster down further to higher-resolution GC levels, two Australian *S.* Enteritidis isolates, which were collected in April 2018, were of the same GC2 cluster as the three isolates from the United Kingdom, the United States, and Ireland, were also found in 2018 (unknown year for the United States isolate). The data suggest a potential international outbreak cluster transmitted to Australia. Other isolates with different GC2 cluster types were collected from 2004 to 2017. Notably, according to the publicly available metadata of source types, GC10-C89 included 24 invasive infection isolates which were observed in South Africa, Australia, and the United States (Fig. 8). The interactive version of the GC10-C89 dendrogram and the linked spatial, temporal, and metadata information of Fig. 8 are available online in Microreact (https://microreact.org/project/6y1Qay9BRdvZBdSGTWzfHr).

### Presence of AMR genes in Australian *S.* Enteritidis.

Antimicrobial resistance is emerging in *S.* Enteritidis, especially in the two lineages prevalent in Africa (2). We evaluated the presence of AMR genes in the Australian *S.* Enteritidis genomes. No AMR genes were observed in isolates from clades A and C. In clade B, 69.6% (500/718) were found to possess AMR genes, and 11.7% (84/718) were predicted to be MDR, with genes conferring resistance to three or more drug classes (Table 3). Among the newly sequenced isolates, 11.6% of clade B isolates were predicted to be MDR. By lineage, 97.6% (82/84) of the MDR isolates belonged to the global lineage MGT4-CC1.

**TABLE 3:** Presence of antimicrobial resistance (AMR) genes in Australian clade B genomes

Presence of AMR genes	No. in all genomes (%)	No. in newly sequenced genomes (%)	No. in publicly available genomes (%)
≥3 drug classes	84 (11.7)	58 (12.3)	26 (10.6)
2 drug classes	82 (11.4)	58 (12.3)	24 (9.8)
1 drug class	334 (46.5)	203 (42.9)	131 (53.5)
No AMR gene	218 (30.4)	154 (32.6)	64 (26.1)
Total	718 (100.0)	473 (100.0)	245 (100.0)

We previously identified MDR-associated STs within which there was a high proportion of predicted MDR genomes (≥80%) at different MGT levels (17). All Australian *S.* Enteritidis genomes (896 isolates) were screened for these MDR-associated STs. There were 47 Australian *S.* Enteritidis isolates found in 10 MDR-associated MGT-STs (Table 4), and 97.9% (46/47) of these isolates were confirmed to carry MDR genes. Therefore, among the 84 Australian clade B genomes carrying MDR genes, 54.8% (46/84) belonged to previously identified MDR-associated MGT-STs. The AMR drug classes of the 10 MDR-associated STs are shown in Table 4; MGT4-ST54, MGT6-ST107, and MGT4-ST354 were the top MDR-associated STs in Australian *S.* Enteritidis.

**TABLE 4:** Multidrug resistance (MDR)-associated MGT-STs in Australian *S.* Enteritidis and the drug classes

MGT-ST	Lineage	No. of Australian isolates	Data for drug classes^*a*^						
			Aminoglycoside	Beta-lactam	Phenicol	Quinolone	Sulphonamide	Tetracycline	Trimethoprim
MGT3-ST15	MGT3-CC15	1	+	+	+		+	+	+
MGT4-ST54	MGT4-CC1	15	+	+		±	+	+	
MGT6-ST107	MGT4-CC1	10	+	+		+	+		
MGT4-ST354	MGT4-CC1	8	+	+		+	+	±	
MGT3-ST9	MGT4-CC1	4	+	+		±	+	+	
MGT3-ST435	MGT4-CC1	3	+	+		+	+	±	
MGT5-ST86	MGT4-CC1	3	+	+		+	+		
MGT4-ST955	MGT4-CC1	1	+	+		+	+		
MGT4-ST1419	MGT4-CC1	1	+	+		+	+		
MGT5-ST2729	MGT4-CC1	1	+	+		+	+		

a The drug classes were predicted using ResFinder (64). +, ≥80% of the isolates were predicted to be resistant to the drug class; ±, <80% but ≥20% of isolates were predicted to be resistant to the drug class.

Plasmids were associated with the acquisition of AMR genes. We screened the Australian *S.* Enteritidis genomes for plasmid replicon types. In clade A, only 10.9% (16/147) of the isolates had plasmid replicon genes (10 types), with IncI1 as the most common type observed in eight isolates (Table S2). In clade C, 4 out of 31 isolates (12.9%) had plasmid replicon genes observed belonging to four different types. In clade B, 32 plasmid replicon types were found in one or more clade B genomes, with 97.5% (700/718) of isolates carrying at least one replicon gene (Table S2). Except for IncFII(S) and IncFIB(S), which were present in >94% of the clade B isolates, IncX1_4 was the dominant type (22.1%), followed by IncX1 (12.1%) and Col156 (11.0%). We further estimated the presence of AMR genes in isolates possessing different plasmid replicon types (Fig. S5). Except for IncFII(S) and IncFIB(S), more than 85% of the isolates with IncX1 (IncX1_1 or IncX1_4) plasmid types were found to carry AMR genes (Fig. S5). Nearly 40% of isolates with IncX1_4 plasmids were predicted to be MDR.

## DISCUSSION

In Australia, *S.* Enteritidis is the second most common serovar after *S.* enterica serovar Typhimurium causing human nontyphoidal salmonellosis; however, *S.* Enteritidis appears to be not prevalent in Australian farms (18–20). Most *S.* Enteritidis infections have been associated with travel, and there were relatively few locally acquired cases (18–20, 30). Therefore, the molecular epidemiology of *S.* Enteritidis in Australia has been significantly different from locally prevalent Salmonella enterica serovars and distinct from that in other countries. To investigate the genomic epidemiology of Australian *S.* Enteritidis, this study characterized 896 Australian genomes (568 were newly sequenced) by comparing them with ~40,000 international genomes in the MGTdb. All Australian *S.* Enteritidis isolates belonged to clade A, B, or C, with clade B being dominant (80.1%), consistent with a previous report (15). An additional two small clades were recently identified from global data (16), but neither was found in Australian *S.* Enteritidis.

### Australian clades A and C are rarely linked to international isolates, and clade A can cause small-scale local outbreaks.

In the global *S.* Enteritidis MGTdb, more than 85% of clade A and 70% of clade C genomes were from Australia. Using seven-gene MLST (MGT1), clade A includes two STs and clade C includes only one. MGT1-ST3304 (clade A) was not observed in any other country, as was previously reported (15). MGT1-ST180 (clade A) was also found in New Zealand, Vanuatu, Vietnam, Germany, the United Kingdom, and the United States. MGT1-ST1972 (clade C) was also found in the United Kingdom and the United States. Within Australia, clade A was significantly more common in QLD than in NSW, and clade C was observed only in QLD. This agrees with a previous report that QLD had the largest number of locally acquired *S.* Enteritidis infections (19). Factors that affect the differences in the distribution of the two clades between states in Australia are unknown.

Using GCs and a 4-week window for clustered isolates as potential outbreaks, the data showed a few small clusters that were potentially outbreak clusters, although there was no epidemiological information to confirm them. For clade A, there were multiple small GC2 clusters with 2 to 8 isolates each within clade A that included isolates collected within 4 weeks, representing more than 40% of all newly sequenced clade A isolates. The largest cluster at GC2 included 8 isolates and was found in both NSW and QLD within 4 weeks in 2018, suggesting a likely outbreak. Therefore, clade A has the potential to cause small-scale outbreaks. This was supported by a previous local outbreak in QLD caused by phage type 26 (22), which belongs to clade A. Clade A isolates were historically found in samples related to chickens, eggs, dogs, and barramundi in QLD (15). Therefore, the environmental reservoir of clade A is likely to be diverse. Investigations of the food/environmental sources of such small clusters in the future would help confirm such potential small-scale outbreaks as well as prevent large-scale clade A outbreaks. In contrast, no GC2 clusters were observed for clade C, with only one cluster of two isolates found at GC5 in QLD. Thus, clade C mainly causes sporadic infections in QLD in Australia.

### Most Australian clade B isolates were likely to have been imported.

Clade B is a global epidemic clade, which includes at least 10 lineages as defined in our previous study (17). Seven lineages were observed in Australia, with MGT4-CC1 as the dominant lineage (>80%), followed by MGT4-CC30. MGT4-CC1 is the global epidemic lineage, which corresponds to the global epidemic clade/lineage in the Feasey et al. (2) and Li et al. studies (31) and the HC100_87 cluster in Achtman et al. (16). The global epidemic lineage (MGT4-CC1) included phage type 4 (PT4) strains (31) and is prevalent in all continents except North America (17). Lineage MGT4-CC30 was mainly observed in Europe (17). Because of the large number of isolates from the NSW outbreak cluster, MGT4-CC30 was ranked as the second-largest lineage of Australian *S.* Enteritidis. Excluding the outbreak isolates, the European lineage MGT4-CC30 is rare in Australia, with only six isolates. Interestingly, three isolates obtained from extraintestinal sources (blood, wound, and ankle fluid) belonged to the two African MDR and invasive lineages (represented by MGT3-CC10 and MGT3-CC15) (2).

Australian clade B isolates from NSW and QLD were found to be frequently linked to isolates from other countries using higher-resolution typing levels (MTG4 or above and GCs at the MGT9 level). In the MGTdb, although there were a limited number of genomes from Asian countries, three out of five top MGT5-STs (all belonging to the MGT4-CC1 lineage) in Australia were also top STs in Asia, suggesting that the Australian *S.* Enteritidis may be epidemiologically linked with Asian countries (Table 2). By GC, 58.1% and 82.2% of Australian clade B isolates belonged to international clusters at GC5 and GC10, respectively (Fig. S2). These findings were supported by data from the annual reports of OzFoodNet; 75% to 95% of *S.* Enteritidis infections were travel related from 2005 to 2011, and more than 80% of travel-related *S.* Enteritidis infections were acquired from Southeast Asia (mainly Bali, Thailand, and Malaysia) (18–20, 30), which are popular Australian tourist destinations. However, clade B isolates were also likely to be imported from other continents. For example, the European and North American prevalent lineage (MGT4-CC13) was also observed in Australia, although less frequently. Overall, by comparison with international isolates using MGT, the majority of Australian clade B genomes were mainly linked to isolates from Asia, followed by Europe.

Apart from international travel-related infections, there were also potential small outbreaks or transmissions within Australia. At the MGT9-ST, GC1, and GC2 levels, 16.5%, 27.5%, and 34.7%, respectively, of Australian clade B isolates from NSW and QLD were grouped together in MGT9 ST or GC clusters and were isolated within a 4-week window. These small clusters may be caused by a common infection source overseas, local secondary transmissions, or consumption of the same imported contaminated food. The transmission from local farms to humans is less likely considering that *S.* Enteritidis remains uncommon in the Australian poultry industry (32, 33). These findings highlight the need for further investigations, as travel history and food exposure metadata were not available in this data set, that could help to better understand the transmission pathways in small closely related clusters and to monitor possible local sources of *S.* Enteritidis causing outbreaks.

### Imported clade B can establish itself locally and cause outbreaks in Australia.

The global spread of *S.* Enteritidis can be mediated by the trade of poultry and poultry feeds. The study by Li et al. found that poultry trade was the main route of spread (31). Australia banned large-scale import of poultry from for several decades starting in the 1930s (34). Only in the late 1980s and early 1990s were imports permitted under strict quarantine (35). All imported breeds entered as hatching eggs and were quarantined in government- or industry-owned quarantine facilities (35), with strict biosecurity practices (36). Australia also prohibits imports of grains as poultry feeds with exceptions during shortages and under the Australian Quarantine and Inspection Service supervision (35). These biosecurity measures must have prevented the globally distributed clade B *S.* Enteritidis from entering Australian farms through poultry-associated trade. Additionally, the strict Australian farm biosecurity measures (36) have successfully prevented the transmission of this pathogen from imported human cases of *S.* Enteritidis infection to Australian poultry farms. Thus, *S.* Enteritidis was not a major cause of local poultry-associated outbreaks in Australia for decades (21).

However, the large outbreak in Australia is an alarming lesson that travel-related or imported strains can potentially colonize local farms and cause large outbreaks, despite stringent biosecurity protection against Salmonella in Australia (37). The egg-associated NSW outbreak caused by *S.* Enteritidis in 2018 is the first large-scale local *S.* Enteritidis outbreak recorded in Australia (23, 24). This outbreak caused more than 200 cases and spread to other Australian states (e.g., QLD and Victoria) and New Zealand (23, 24). This study investigated this large local outbreak by comparing isolates with international strains in the global MGT database. We found that three isolates from the United Kingdom and Ireland were closely related (belonging to the same GC5 type) but were unlikely to be directly linked to the Australian outbreak because the three European isolates were collected from 2012 to 2015. By Bayesian analysis, the MRCA of the outbreak isolates was around 2015 (95% CI, 2013 to 2017). The Australian isolates and international isolates (from the United Kingdom) had an MRCA around 2011 (95% CI, 2008 to 2012), suggesting that this outbreak strain may have been imported into Australia around or after 2011, established locally, and then caused a large outbreak.

These findings highlight the potential for travel-related or imported strains to colonize local farms and cause large outbreaks, despite stringent biosecurity protection against Salmonella in Australia (35–37). Therefore, despite the low prevalence of *S.* Enteritidis in Australian farms (21), controlling the spread of imported strains and routine surveillance of clade B *S.* Enteritidis in the food production chain is critical. The establishment of an imported *S.* Enteritidis strain in Australian farms suggests that the absence of *S.* Enteritidis in local farms previously was not due to the farm environment in Australia being unsuitable for *S.* Enteritidis to survive and grow.

Importantly, this outbreak belonged to a recently defined lineage, MGT4-CC30 (17), which was predominantly observed in Europe, and the Australian outbreak is the first large-scale outbreak recorded for this lineage globally. More attention should be paid to this lineage, as it can potentially cause large outbreaks, especially in Europe.

### AMR *S.* Enteritidis in Australia.

MDR *S.* Enteritidis has been highlighted as an emerging threat to human health (2, 38). Clade A and C isolates had no AMR genes identified. This was similar to the earlier study by Graham et al. (15). The rarity of AMR genes in the Australian clades A and C reflected the relatively strict strategies in agricultural usage of antibiotics in Australia (39, 40). In contrast, 69.6% of Australian clade B isolates possessed at least one AMR gene, and 11.7% of clade B isolates were predicted to be MDR. Using STs at different MGT levels, 54.8% of the MDR isolates belonged to previously identified MDR-associated MGT STs (17), with MGT4-ST54, MGT6-ST107, and MGT4-ST354 as the major MDR-associated STs. Interestingly, all these STs belonged to MGT3-ST8, which was one of the dominant types in Asian countries such as China (41, 42), South Korea, and Thailand. These findings further confirmed that clade B *S.* Enteritidis strains isolated in Australia were likely to have originated from other countries, likely from Asia, as part of the global spread of these MDR clones. Continuous surveillance of MDR *S.* Enteritidis is critical because MDR-associated infections are an increasing threat (2, 43–45). MGT STs are potential good candidates for the global unified tracking of MDR clones. With more genomes available from countries where there is more antibiotics overuse or misuse, more MDR-associated MGT STs are expected to be identified and their origins identified.

Plasmids are the main mechanism for the acquisition of AMR genes (46). IncX1 plasmids (IncX1_4 and IncX1_1) were the most common plasmid replicon types in Australian clade B genomes, and more than 80% of isolates with these plasmid types harbored AMR genes. The predicted MDR rate in isolates with plasmid IncX1_4 was nearly 40%, similar to our previous findings that a significantly higher proportion of isolates with IncX1_4 carried MDR genes (17). Graham et al. also found that the IncX1 plasmid was common in clade B *S.* Enteritidis that carried AMR genes (15). An IncX1 plasmid with 16 AMR genes had been reported in Salmonella enterica serovar Agona from Australia (47). Therefore, more attention should be paid to isolates with plasmid replicon type IncX1 (especially IncX1_4), which may have a higher risk of MDR acquisition and transmission.

### The application of MGT to epidemiological surveillance of *S.* Enteritidis.

Public health laboratories across Australia have initiated genome-based surveillance within the communicable disease genomics network (CDGN), and a national microbial genomics framework has been formulated (48). The Australian national genomics framework has outlined the following key requirements for the integration of genomic surveillance in public health systems (49): (i) establishment of standardized national microbial genomic surveillance approaches using unified nomenclatures, (ii) development of standardized genomic analysis reporting outputs for public health surveillance and response, (iii) rapid and sustainable data sharing between public health laboratories, and (iv) an open-source platform for data processing and data repository.

The global open MGTdb for *S.* Enteritidis is a promising tool that can cater to all of these requirements. (i) MGT uses standardized and consolidated nomenclature (STs/CCs of MGT) to identify predefined clades/lineages of *S.* Enteritidis, depict the geographic and temporal epidemiology using MGT STs, identify important clones (e.g., the MDR-associated MGT STs), and investigate potential outbreak clusters using the scalable-resolution GCs. Thus, MGT enables standardized national and international microbial genomic surveillance. (ii) MGT consists of multiple allele-by-allele comparison-based MLST schemes (25), which does not require the construction of a phylogenetic tree and is computationally rapid. By describing the relationships of isolates using the numeric STs and cluster types at different MGT levels, the data interpretation could be simple and fast. To further simplify the data interpretation, the online platform allows users to select and visualize the epidemiological distribution of STs/CCs at different MGT levels (https://mgtdb.unsw.edu.au/enteritidis/summaryReport) (50). (iii). An open database for *S.* Enteritidis is available (https://mgtdb.unsw.edu.au/enteritidis/), which allows for unified typing among different laboratories and comparison with publicly available international genomes. In summary, the comparison of the genomic features between states and countries using MGT offered a more comprehensive picture to understand *S.* Enteritidis in Australia. MGT is a promising system for surveillance of *S.* Enteritidis in Australia and is a good candidate to be integrated into the public health surveillance system.

### Challenges of using public genome data.

There are two challenges related to the use of publicly available genomes that are relevant to this study. First, genome sequence data sets from different continents can be biased, with Asia, South America, and Africa being underrepresented. This means that international clusters were only described if they happened to be found in Australia and a region with better coverage of WGS data (North America/Europe). Second, global genomic surveillance relies on the availability and accuracy of metadata. Exact collection dates could facilitate more accurate outbreak investigations, and sources of samples or types of infections (e.g., gastroenteritis or iNTS) would facilitate the risk assessment of imported *S.* Enteritidis.

### Conclusion.

This analysis of a large representative set of *S.* Enteritidis genomes using the MGT scheme illustrated the scalable resolution of MGT typing and demonstrated that Australian *S.* Enteritidis belonged to clades A, B, and C. Clades A and C were confirmed to be endemic in Australia and were not linked to international clusters. However, clade A may have caused small-scale (2 to 12 isolates) local outbreaks. Clade B isolates were dominated by the global epidemic lineage MGT4-CC1 and likely mostly travel related. The locally acquired egg-associated clade B outbreak cluster was found belonging to a lineage (MGT4-CC30) prevalent in Europe. AMR genes were only observed in clade B genomes and were especially common in isolates with plasmid type IncX1. *S.* Enteritidis MGT provides a systematic and sharable nomenclature and platform that can be effectively applied to public health agencies for unified surveillance of *S.* Enteritidis globally. The application of the *S.* Enteritidis MGT platform to the Australian *S.* Enteritidis population in two states of Australia showcased its value for high-resolution epidemiological surveillance in Australia and internationally.

## MATERIALS AND METHODS

### Source of isolates.

A total of 613 clinical *S.* Enteritidis isolates from 2017–2018 from QLD and NSW were sequenced as part of routine public health laboratory surveillance. Raw reads were processed using the same methods as in our previous study (17), including contamination detection (Kraken v0.10.5) (51), quality filtering (Quast v5.0.2) (52, 53), assembly (SKESA v2.3) (54), serotyping (SISTR v1.0.2) (54), multilocus sequence typing (MLST) (1), MGT loci extraction, and MGT calling (25). A total of 568 newly sequenced *S.* Enteritidis genomes passed quality filters and were successfully processed by MGT. There were 36 (6.3%) poor-quality genomes and four non-*S.* Enteritidis genomes. Isolates from the updated global MGTdb for *S.* Enteritidis included 40,390 genomes that were released to be publicly available by April 2021 (including 40,062 non-Australian and 328 Australian *S.* Enteritidis genomes, excluding duplicates).

### Population structure elucidation of the Australian *S.* Enteritidis isolates.

Our previous study described clades and lineages for *S.* Enteritidis using MGT STs and clonal complexes (CCs, single-linkage clustering STs with one allele difference) at different MGT levels (17). The clades and lineages were assigned to the Australian *S.* Enteritidis genomes (896 in total) using these predefined clade/lineage markers (MGT1 to MGT5 STs/CCs) using custom Python scripts (https://github.com/Adalijuanluo/MGTSEnT).

### Identification and visualization of clusters of closely related isolates.

Closely related isolates were grouped into genomic clusters (GCs) at multiple cutoffs (i.e., 1-, 2-, 5-, and 10-allele differences) based on the highest resolution level, MGT9. Note that GCs are equivalent to our previously defined ODCs (25). In this study we used GCs to avoid confusion with clusters of epidemiologically confirmed outbreaks. The average number of isolates in each cluster increased as the allowed allele differences were increased from 1 to 10 (for GC1 to GC10).

From MGT9 STs to GC10 clusters, any STs or GCs with two or more isolates were identified using custom scripts. The characteristics of these clusters across the country, state, and periods were depicted using Tableau v2019.2 (55). Singletons refer to cluster types with only one isolate each in the sample. A cluster that could be potentially linked to an outbreak was defined as a nonsingleton cluster with at least two isolates recovered from samples collected within a 4-week window.

Microreact (29) was used to visualize clusters at different GC levels. A dendrogram of all isolates of a GC10 cluster was constructed to visualize the division of clusters by sequentially lower GC levels. For a single GC10 cluster of interest (a GC10 cluster includes potential outbreak clusters), clusters at GC1 to 9 were recalled within the GC10 cluster. Note that the MGTdb only includes GC1 (MGT9 CC), 2, 5, and 10. Here, we also included GC3, 4, 6, 7, 8 and 9, to increase the flexibility of outbreak clusters selection. A dendrogram was produced based on the recalled cluster types from GC1 to GC10, as well as MGT9 STs. The root was the GC10 cluster, each leaf was an MGT9 ST, and each internal node represented a GC cluster between GC1 and GC9. Therefore, the dendrogram is a GC typing tree rather than a phylogenetic tree. The dendrogram and the metadata were visualized in Microreact (29). Isolates with invasive infections can be highlighted in Microreact (29). The geographic distribution and isolation timeline of different subclusters can be visualized and compared in Microreact (29).

### Bayesian evolutionary analysis of the Australian outbreak lineage.

Bayesian evolutionary analysis was performed to investigate the evolutionary history of the isolates associated with the Australian outbreak. For the isolates belonging to the European common lineage, 144 isolates have unique MGT8 STs which were sampled for phylogenetic tree construction using quicktree.pl (which is a pipeline included in SaRTree v1.2.2) with P125109 as reference (56). Ten sampled Australian outbreak isolates were phylogenetically close to 18 international isolates and one nonoutbreak Australian isolate, which belonged to the same branch. These 29 isolates were selected to estimate the evolutionary history of the large-scale egg-associated outbreak in Australia; Bayesian evolutionary analysis was performed using BEAUti v1.10.4 and BEAST v1.10.4 (57). Eight models were evaluated with the general time-reversible model (GTR) gamma model, Markov chain Monte Carlo (MCMC) chain length of 100,000,000, and a phylogenetic expansion effective sample size (ESS) of >200 (57). The population expansion was estimated using Tracer v1.7.1 with 10% burn-in (58). Single-nucleotide polymorphism (SNP) alignments were produced from the MGT9 allele profile. RecDetect v1.2.2 (56) was used to remove the recombination sites on the SNP alignments. A maximum likelihood tree (GTR, gamma model) was constructed using MEGA X (59) with a bootstrap number of 1,000. The log file of the Bayesian skyline model was mapped onto the maximum likelihood tree using the BEAST tree annotator v1.10.4 and visualized using Figtree v1.4.4 (57, 60).

### AMR prediction and plasmid replicon genes analysis.

AMR-associated genes and mutations were screened using the AMRFinderPlus v3.10.18 database using the default settings (61, 62). Our previous study (17) estimated the presence of AMR genes in all publicly available genomes of *S.* Enteritidis and identified MGT-STs that were associated with MDR using custom Python scripts. These MDR-associated STs were identified among the Australian *S.* Enteritidis genomes. In addition, plasmid replicon genes were screened against the PlasmidFinder v2.1 database (63) through Abricate v1.0.1 (https://github.com/tseemann/abricate). Plasmid replicon genes with identity and coverage of ≥90% were regarded as positive.

### Data availability.

All newly sequenced data in this work were deposited in the National Center for Biotechnology Information (NCBI) under BioProjects PRJNA489746 and PRJNA848119. The detailed metadata are available in MGTdb at https://mgtdb.unsw.edu.au/enteritidis/.
